# Supplementary material for: Risk factors associated with SARS-CoV-2 infection in a multiethnic cohort of United Kingdom healthcare workers (UK-REACH): A cross-sectional analysis
Source: PLoS Med. 2022 May 26;19(5):e1004015. doi: 10.1371/journal.pmed.1004015 (PMC9187071; doi:10.1371/journal.pmed.1004015)
Supplement: S1 Table — COVID-19, Coronavirus Disease 2019; PCR, polymerase chain reaction. (DOCX) [file pmed.1004015.s003.docx]

**S1 Table. Relative contribution of PCR, serology and suspected COVID-19 to overall number of infections in both cohorts**

| **Condition** | **Number (proportion) meeting condition**  **Excluding those not working during lockdown (n=10,772)** | **Number (proportion) meeting condition**  **Including those not working during lockdown (n=12,541)** |
| --- | --- | --- |
| **PCR positive (negative, not tested or missing for serology)** | 1013 (9.4%) | 1167 (9.3%) |
| **Serology positive (negative, not tested or missing for PCR)** | 899 (8.3%) | 964 (7.7%) |
| **PCR and serology positive** | 412 (3.8%) | 436 (3.5%) |
| **Not tested by PCR or serology (or both missing) but suspected COVID-19** | 172 (1.6%) | 224 (1.8%) |
| **Total infections** | 2496 (23.2%) | 2791 (22.3%) |

COVID-19 – coronavirus disease 2019, n – number, PCR – polymerase chain reaction
