# Supplementary material for: Risk factors associated with SARS-CoV-2 infection in a multiethnic cohort of United Kingdom healthcare workers (UK-REACH): A cross-sectional analysis
Source: PLoS Med. 2022 May 26;19(5):e1004015. doi: 10.1371/journal.pmed.1004015 (PMC9187071; doi:10.1371/journal.pmed.1004015)
Supplement: S2 Table — (DOCX) [file pmed.1004015.s004.docx]

**S2 Table. Derivation of covariates from questionnaire data**

| **Variable** | **Description** |
| --- | --- |
| **Age** | Continuous variable. Age in years. Derived from date of birth entered by participants at registration. |
| **Sex** | Binary variable. Participants were asked their sex assigned at birth. |
| **Ethnicity** | Categorical variable. Participants were asked to select their ethnicity from a list of the 18 Office for National Statistics categories:  Asian/Asian British – Indian  Asian/Asian British – Pakistani  Asian/Asian British – Bangladeshi  Asian/Asian British – Chinese  Asian/Asian British - Any other Asian background  Black/African/Caribbean/Black British - African  Black/African/Caribbean/Black British – Caribbean  Black/African/Caribbean/Black British - Any other Black/African/Caribbean background Mixed/Multiple ethnic groups - White and Black Caribbean  Mixed/Multiple ethnic groups - White and Black African  Mixed/Multiple ethnic groups - White and Asian  Mixed/Multiple ethnic groups - Any other Mixed/multiple ethnic background  White - English/Welsh/Scottish/Northern Irish/British  White – Irish  White - Gypsy or Irish Traveller  White - Any other white background  Other ethnic group – Arab  Other ethnic group - Any other ethnic background  These were categorised into the 5 broader Office for National Statistics ethnicity categories (Asian, Black, Mixed, White, Other). |
| **Migration status** | Binary variable. Participants were asked whether they were born in the UK. |
| **Religiosity** | Ordinal variable. Participants were asked “How important is religion to you in your everyday life?” and could answer using the following scale: not at all important, fairly important, very important, extremely important and prefer not to answer. This question was asked only to those who indicated that they identified as belonging to a particular religious group in a previous question. Those who indicated they had “no religion” were grouped together with those indicating religion was not at all important. |
| **Household size** | Continuous variable. Participants were asked how many people live in their house other than themselves. |
| **Cohabitation (with key workers)** | Binary variable. Participants were asked “Apart from yourself, how many people in your household work in jobs that often bring them into close physical contact (within 2 metres) with others? Some examples include: bus driver, carer, cleaner, doctor, supermarket checkout worker, teacher.”. This was categorised into the binary ‘does not live with another key worker’ vs ‘lives with another key worker’. |
| **Accommodation (contains shared spaces)** | Binary variable. Participants were asked to answer yes or no to the question “Does your accommodation include shared communal areas such as hallways, stairwells or lifts?” |
| **Index of Multiple Deprivation (IMD) quintile** | Ordinal variable. Participants provided their residential postcode on registration for the study. This was used to determine the Index of Multiple Deprivation (the official measure of deprivation for small areas of England) in the area in which they live. The IMD ranks all areas in England based on 7 measures of deprivation and the ranks can be expressed as quintiles. Lower quintiles indicate more deprivation. Although Wales, Scotland and Northern Ireland have their own measures of deprivation, these are said not to be directly comparable to English IMD and therefore we elected to impute an ‘English IMD’ for residents of the these nations. |
| **Social mixing** | Categorical variable. Participants were asked to indicate the number of contacts they had with other outside of their work both face-to-face with social distancing and with physical contact. Answers were used to derive a three level categorical variable: “No social contact or all remote”, “social contact but socially distanced”, “social contact with physical contact”. |
| **Comorbidities (diabetes and immunosuppression)** | Binary variables. Participants were asked to indicate if they had “diabetes (type I or II)” or “ A weakened immune system or reduced ability to deal with infections (as a result of a disease or treatment)” |
| **Shielding status** | Binary variable. Participants were asked “Have you been contacted by letter or text message to say you are at severe risk from COVID-19 due to an underlying health condition and should be shielding?” |
| **Smoking status** | Binary variable. Participants were asked to indicate their current smoking status. Never and ex-smokers were grouped together and compared with current smokers. |
| **COVID-19 vaccination status** | Binary variable. Participants were coded as vaccinated if they indicated they had received at least one dose of COVID-19 vaccine at the point at which they responded to the baseline questionnaire. |
| **Occupation** | Categorical variable. Participants were asked to select their main job/role. Categorised as below:  **Doctor or medical support** - Doctor, Advanced Critical Care Practitioner, Anaesthesia associate, Surgical Care Practitioner, Other medical associate  **Nurse, NA or Midwife -**  Advanced Nurse Practitioner, Healthcare assistant, Maternity support worker, Midwife, Nurse, Nursing Associate, Other nursing and midwifery role,  **Allied Health Professional (including pharmacists, ambulance workers and those in optical roles)** - Arts therapist, Biomedical scientist, Chiropodist/Podiatrist, Clinical scientist, Dietician, Hearing aid dispenser, Occupational therapist, Operating department practitioner, Orthoptist, Physiotherapist, Practitioner psychologist, Prosthetist / Orthotist, Radiographer, Speech and language therapist, Other Allied Health Professional role, Emergency medical , Paramedic , Other ambulance role, OT Support , Phlebotomist, Physiotherapy Assistant, Radiography Other clinical support role , Pharmacist , Pharmacy technician, Other pharmacy role, Optical - Dispensing optician, Optometrist, Other Optical role  **Dental -**  Clinical dental technician, Dental Hygienist, Dental nurse, Dental technician, Dentist, Other dental role  **Admin, estates or other –** Administration, Catering services, Domestic services, Estates services, Porter, Other |
| **Method of commuting** | Binary variable. Participants were asked the following question: “Which of the following modes of transport do you use to commute on a typical working day over the past month? Please select all that apply” and could select from the following options “Car, alone or with member of household, car share, with a small pool of people outside of household, taxi or private hire vehicle, public transport (e.g. bus, train, tram, underground), motorcycle, scooter or moped, bicycle, on foot, other”. Participants who indicated that at least part of their journey contained a means of transportation that was shared with others outside of their household was coded as “with others outside household”. |
| **Number of SARS-CoV-2 positive patients attended to per week (with physical contact)** | Ordinal variable. Participants were asked to select how many suspected or confirmed COVID-19 patients they attended to (with physical contact) in a week on the following scale: 0, 1-5, 6-20, 21-50, 51+. This was categorised as 0, 1-5, 6 – 20, ≥21. |
| **Access to appropriate PPE** | Binary variable. Participants were asked to indicate how frequently they had access to PPE in the first month after the start of UK national lockdown using the following scale “Not applicable, not at all, rarely, some of the time, yes, most of the time, yes, all of the time. This was collapsed into the binary variable “not applicable or all or most of the time” vs “some of the time or less frequently” |
| **Work areas** | Binary (dummy) variables. Participants were asked to select areas in which they work from a list of non-mutually exclusive clinical and non-clinical areas. |
| **Aerosol generating procedure exposure** | Binary variable. Derived from a question that asked how often participants were in a room where aerosol generating procedures are performed. On the following scale: Not applicable, Never, Once a month or less, A few times a month, Once a week, A few times a week, Every day. |
| **Night shift pattern** | Categorical variable. Participants were asked how often they work night shifts. On the following scale: Not applicable, Never, Less than once a month, Once a month or more, but not every week, Once a week or more, but not every shift, I always work nights. This was categorised as never, less than weekly or weekly/always |
| **Work region** | Categorical variable. Participants were asked to enter the first part of the postcode of their place of work. This was mapped to one of 12 UK regions. |
