# Supplementary material for: Risk factors associated with SARS-CoV-2 infection in a multiethnic cohort of United Kingdom healthcare workers (UK-REACH): A cross-sectional analysis
Source: PLoS Med. 2022 May 26;19(5):e1004015. doi: 10.1371/journal.pmed.1004015 (PMC9187071; doi:10.1371/journal.pmed.1004015)
Supplement: S3 Table — IQR, interquartile range; med, median; NA, nursing associate; PPE, personal protective equipment. (DOCX) [file pmed.1004015.s005.docx]

**S3 Table. Description of the cohort working during lockdown stratified by ethnicity together with tests of association between predictor variables and ethnicity**

| **Variable** | **Whole cohort working during lockdown (n=10,722)** | **White** | **Asian** | **Black** | **Mixed** | **Other** | **P value** |
| --- | --- | --- | --- | --- | --- | --- | --- |
| **Age**, med(IQR)  Missing | 45 (35 – 54)  54 (0.5%) | 46 (36 – 55)  35 (0.5%) | 42 (33 – 51)  12 (0.6%) | 43.5 (34.5 – 54)  2 (0.4%) | 41 (32 – 50)  3 (0.7%) | 43 (35 – 53)  2 (0.9%) | <0.001 |
| **Sex**  Male  Female  Missing | 2660 (24.7%)  8089 (75.1%)  23 (0.2%) | 1485 (19.6%)  6085 (80.3%)  13 (0.2%) | 813 (39.5%)  1241 (60.3%)  3 (0.2%) | 149 (32.3%)  311 (67.3%)  2 (0.4%) | 105 (23.5%)  339 (76.0%)  2 (0.5%) | 108 (48.2%)  113 (50.5%)  3 (1.3%) | <0.001 |
| **Migration status**  Born in UK  Born abroad  Missing | 7901 (73.5%)  2847 (26.5%)  24 (0.2%) | 6642 (87.6%)  926 (12.2%)  15 (0.2%) | 734 (35.7%)  1317 (64.0%)  6 (0.3%) | 144 (31.2%)  316 (68.4%)  2 (0.4%) | 340 (76.2%)  106 (23.8%)  0 (0.0%) | 41 (18.3%)  182 (81.3%)  1 (0.5%) | <0.001 |
| **Religiosity**  Not religious /not important  Fairly important  Very important  Extremely important  Missing | 6085 (56.5%)  2268 (21.1%)  1064 (9.9%)  1124 (10.4%)  231 (2.1%) | 5041 (66.5%)  1468 (19.4%)  492 (6.5%)  439 (5.8%)  143 (1.9%) | 624 (30.3%)  613 (29.8%)  373 (18.1%)  390 (19.0%)  57 (2.8%) | 68 (14.7%)  77 (16.7%)  118 (25.5%)  190 (41.1%)  9 (2.0%) | 288 (64.6%)  73 (16.4%)  29 (6.5%)  42 (9.4%)  14 (3.1%) | 64 (28.6%)  37 (16.5%)  52 (23.2%)  63 (28.1%)  8 (3.6%) | <0.001 |
| **Household size,** med (IQR)  Missing | 2 (1 – 3)  8 (0.1%) | 2 (1 – 3)  3 (0.0%) | 2 (1 – 3)  3 (0.2%) | 2 (1 – 3)  0 (0.0%) | 2 (1 – 3)  1 (0.2%) | 2 (1 – 3)  1 (0.5%) | <0.001 |
| **Cohabitation**  Does not live with other key workers  Lives with other key workers  Missing | 5571 (51.7%)  5145 (47.8%)  56 (0.5%) | 4025 (53.1%)  3527 (46.5%)  31 (0.4%) | 970 (47.2%)  1073 (52.2%)  14 (0.7%) | 211 (45.7%)  246 (53.3%)  5 (1.1%) | 246 (55.2%)  197 (44.2%)  3 (0.7%) | 119 (53.1%)  102 (45.5%)  3 (1.3%) | <0.001 |
| **Accommodation**  Does not have shared spaces  Has shared spaces  Missing | 8807 (81.8%)  1905 (17.7%)  60 (0.6%) | 6491 (85.6%)  1054 (13.9%)  38 (0.5%) | 1492 (72.5%)  552 (26.8%)  13 (0.6%) | 327 (70.8%)  133 (28.8%)  2 (0.4%) | 345 (77.4%)  99 (22.2%)  2 (0.5%) | 152 (67.9%)  67 (29.9%)  5 (2.2%) | <0.001 |
| **Index of multiple deprivation quintile**  1 (most deprived)  2  3  4  5 (least deprived)  Missing | 956 (8.9%)  1597 (14.8%)  1944 (18.1%)  2312 (21.5%)  2700 (25.1%)  1263 (11.7%) | 592 (7.8%)  1060 (14.0%)  1375 (18.1%)  1643 (21.7%)  1934 (25.5%)  979 (12.9%) | 199 (9.7%)  337 (16.4%)  339 (16.5%)  477 (23.2%)  523 (25.4%)  182 (8.9%) | 89 (19.3%)  97 (21.0%)  95 (20.6%)  65 (14.1%)  79 (17.1%)  37 (8.0%) | 41 (9.2%)  69 (15.5%)  96 (21.5%)  84 (18.8%)  117 (26.2%)  39 (8,7%) | 35 (15.6%)  34 (15.2%)  39 (17.4%)  43 (19.2%)  47 (21.0%)  26 (11.6%) | <0.001 |
| **Social mixing**  None or all remote  Face to face (with social distancing)  Physical contact  Missing | 2685 (25.0%)  6584 (61.4%)  1460 (13.6%)  43 (0.4%) | 1659 (21.9%)  4874 (64.3%)  1023 (13.5%)  27 (0.4%) | 710 (34.5%)  1095 (53.2%)  242 (11.8%)  10 (0.5%) | 142 (30.7%)  243 (52.5%)  75 (16.2%)  2 (0.4%) | 107 (24.0%)  256 (57.4%)  80 (17.9%)  3 (0.7%) | 67 (29.9%)  116 (51.8%)  40 (17.9%)  1 (0.5%) | <0.001 |
| **Comorbidities**  Not diabetic  Diabetic  Missing | 9918 (92.1%)  400 (3.9%)  454 (4.2%) | 7053 (93.0%)  225 (3.0%)  305 (4.0%) | 1840 (89.5%)  125 (6.1%)  92 (4.5%) | 412 (89.2%)  24 (5.2%)  26 (5.6%) | 411 (92.2%)  18 (4.0%)  17 (3.8%) | 202 (90.2%)  8 (3.6%)  14 (6.3%) | <0.001 |
| **Comorbidities**  Not Immunosuppressed  Immunosuppressed  Missing | 9983 (92.7%)  335 (3.1%)  454 (4.2%) | 7023 (92.6%)  255 (3.4%)  305 (4.0%) | 1917 (93.2%)  48 (2.3%)  92 (4.5%) | 427 (92.4%)  9 (2.0%)  26 (5.6%) | 414 (92.2%)  15 (3.4%)  17 (3.8%) | 202 (90.2%)  8 (3.6%)  14 (6.3%) | 0.09 |
| **Shielding status**  Not advised to shield  Advised to shield  Missing | 10,324 (95.8%)  410 (3.8%)  38 (0.4%) | 7301 (96.3%)  256 (3.4%)  26 (0.3%) | 1960 (95.3%)  89 (4.3%)  8 (0.4%) | 429 (92.9%)  31 (6.7%)  2 (0.4%) | 425 (95.3%)  20 (4.5%)  1 (0.2%) | 209 (93.3%)  14 (6.3%)  1 (0.5%) | 0.008 |
| **Smoking status**  Never/ex-smoker  Current smoker  Missing | 10,139 (94.1%)  533 (5.0%)  533 (5.0%) | 7106 (93.7%)  426 (5.6%)  51 (0.7%) | 1964 (95.5%)  61 (3.0%)  32 (1.6%) | 448 (97.0%)  9 (2.0%)  5 (1.1%) | 409 (91.7%)  28 (6.3%)  9 (2.0%) | 212 (94.6%)  9 (4.0%)  3 (1.3%) | <0.001 |
| **COVID-19 vaccination status at the time of questionnaire response**  Unvaccinated  Vaccinated  Missing | 3853 (35.8%)  4939 (45.9%)  1980 (18.4%) | 2697 (35.6%)  3325 (43.9%)  1561 (20.6%) | 705 (34.3%)  1104 (53.7%)  248 (12.1%) | 196 (42.4%)  203 (43.9%)  63 (13.6%) | 165 (37.0%)  201 (45.1%)  80 (17.9%) | 90 (40.2%)  106 (47.3%)  28 (12.5%) | <0.001 |
| **Occupation**  Doctor or medical support  Nurse, NA or Midwife  Allied Health Professional*  Dental  Admin, estates or other  Missing | 2596 (24.1%)  2354 (21.9%)  4422 (41.1%)  418 (3.9%)  607 (5.6%)  375 (3.5%) | 1045 (13.8%)  2026 (26.7%)  3503 (46.2%)  290 (3.8%)  494 (6.5%)  225 (3.0%) | 1092 (53.1%)  159 (7.7%)  550 (26.7%)  96 (4.7%)  70 (3.4%)  90 (4.4%) | 170 (36.8%)  90 (19.5%)  152 (32.9%)  11 (2.4%)  13 (2.8%)  26 (5.6%) | 164 (36.8%)  55 (12.3%)  176 (39.5%)  14 (3.1%)  22 (4.9%)  15 (3.4%) | 125 (55.8%)  24 (10.7%)  41 (18.3%)  7 (3.1%)  8 (3.6%)  19 (8.5%) | <0.001 |
| **Method of commuting**  Alone or with members of household  With others outside household  Missing | 9577 (88.9%)  1061 (9.9%)  134 (1.2%) | 6842 (90.2%)  656 (8.7%)  85 (1.1%) | 1789 (87.0%)  239 (11.6%)  29 (1.4%) | 377 (81.6%)  79 (17.1%)  6 (1.3%) | 390 (87.4%)  48 (10.8%)  8 (1.8%) | 179 (79.9%)  39 (17.4%)  6 (2.7%) | <0.001 |
| **Number of SARS-CoV-2 positive patients attended to per week (with physical contact)**  None  1 – 5  6 – 20  ≥ 21  Missing | 6298 (58.5%)  2169 (20.1%)  1506 (14.0%)  687 (6.4%)  112 (1.0%) | 4702 (62.0%)  960 (12.7%)  440 (5.8%)  162 (7.9%)  68 (0.9%) | 1009 (49.1%)  503 (24.5%)  354 (17.2%)  162 (7.9%)  29 (1.4%) | 229 (49.6%)  118 (25.5%)  78 (16.9%)  30 (6.5%)  7 (1.5%) | 254 (57.0%)  81 (18.2%)  71 (15.9%)  36 (8.1%)  4 (0.9%) | 104 (46.4%)  54 (24.1%)  43 (19.2%)  19 (8.5%)  4 (1.8%) | <0.001 |
| **Access to appropriate PPE**  Not applicable or all/most the time  Some of the time or less frequently  Missing | 4560 (42.3%)  6182 (57.4%)  30 (0.3%) | 3383 (44.6%)  4183 (55.2%)  17 (0.2%) | 713 (34.7%)  1336 (65.0%)  8 (0.4%) | 188 (40.7%)  270 (58.4%)  4 (0.9%) | 186 (41.7%)  259 (58.1%)  1 (0.2%) | 90 (40.2%)  134 (59.8%)  0 (0.0%) | <0.001 |
| **Aerosol generating procedure exposure**  Less than weekly exposure  At least weekly exposure  Missing | 8437 (78.3%)  2296 (21.3%)  39 (0.4%) | 6115 (80.6%)  1449 (19.1%)  19 (0.3%) | 1482 (72.1%)  562 (27.3%)  13 (0.6%) | 342 (74.0%)  115 (24.9%)  5 (1.1%) | 345 (77.4%)  100 (22.4%)  1 (0.2%) | 153 (68.3%)  70 (31.3%)  1 (0.5%) | <0.001 |
| **Night shift pattern**  Never works nights  Works nights less than weekly  Works nights weekly or always  Missing | 7543 (70.0%)  1796 (16.7%)  1317 (12.2%)  116 (1.1%) | 5626 (74.2%)  1090 (14.4%)  799 (10.5%)  68 (0.9%) | 1238 (60.2%)  456 (22.2%)  329 (16.0%)  34 (1.7%) | 271 (58.7%)  100 (21.7%)  81 (17.5%)  10 (2.2%) | 290 (65.0%)  98 (22.0%)  55 (12.3%)  3 (0.7%) | 118 (52.7%)  52 (23.2%)  53 (23.7%)  1 (0.5%) | <0.001 |
| **Work areas**  Ambulance  Community clinical setting / primary care  Non clinical community setting  Emergency Department  Intensive Care Unit  Hospital Inpatient  Hospital Outpatient  Hospital non-clinical area or laboratory  Psychiatric hospital  Maternity  Nursing or Care Home  University  Home  Missing (range) † | 396 (3.7%)  2426 (22.5%)  565 (5.3%)  963 (8.9%)  927 (8.6%)  2759 (25.6%)  1831 (17.0%)  1114 (10.3%)  312 (2.9%)  344 (3.2%)  242 (2.3%)  220 (2.0%)  1715 (15.9%)  34 – 40 (0.3 – 0.4%) | 359 (4.7%)  1731 (22.8%)  462 (6.1%)  536 (7.1%)  619 (8.2%)  1696 (22.4%)  1140 (15.0%)  787 (10.4%)  211 (2.8%)  235 (3.1%)  202 (2.7%)  162 (2.1%)  1249 (16.5%)  19 – 23 (0.3 – 0.3%) | 18 (0.9%)  479 (23.3%)  59 (2.9%)  290 (14.1%)  203 (9.9%)  714 (34.7%)  485 (23.6%)  212 (10.3%)  61 (3.0%)  68 (3.3%)  20 (1.0%)  35 (1.7%)  304 (14.8%)  8 – 11 (0.4 – 0.5%) | $  93 (20.1%)  17 (3.7%)  56 (12.1%)  39 (8.4%)  133 (28.8%)  69 (14.9%)  51 (11.0%)  26 (5.6%)  21 (4.6%)  15 (3.3%)  7 (1.5%)  58 (12.6%)  4 – 5 (0.9 – 1.1%) | 13 (2.9%)  97 (21.8%)  20 (4.5%)  46 (10.3%)  42 (9.4%)  118 (26.5%)  74 (16.6%)  41 (9.2%)  10 (2.2%)  13 (2.9%)  $  8 (1.8%)  72 (16.1%)  0 (0.0%) | $  26 (11.6%)  7 (3.1%)  35 (15.6%)  24 (10.7%)  98 (43.8%)  63 (28.1%)  23 (10.3%)  $  7 (3.1%)  $  8 (3.6%)  32 (14.3%)  2 – 3 (0.9 – 1.3%) | <0.001  0.001  <0.001  <0.001  0.02  <0.001  <0.001  0.31  0.004  0.12  <0.001  0.07  0.04 |
| **Work region**  London  South East England  South West England  East of England  East Midlands  West Midlands  North East England  North West England  Yorkshire and the Humber  Wales  Scotland  Northern Ireland  Missing | 1423 (13.2%)  1265 (11.7%)  857 (8.0%)  759 (7.1%)  1097 (10.2%)  834 (7.7%)  445 (4.1%)  1101 (10.2%)  778 (7.2%)  334 (3.1%)  626 (5.8%)  130 (1.2%)  1123 (10.4%) | 816 (10.8%)  922 (12.2%)  699 (9.2%)  525 (6.9%)  811 (10.7%)  544 (7.2%)  357 (4.7%)  793 (10.5%)  577 (7.6%)  256 (3.4%)  498 (6.6%)  108 (1.4%)  677 (8.9%) | 373 (18.1%)  214 (10.4%)  90 (4.4%)  152 (7.4%)  191 (9.3%)  192 (9.3%)  68 (3.3%)  193 (9.4%)  129 (6.3%)  50 (2.4%)  81 (3.9%)  13 (0.6%)  311 (15.1%) | 107 (23.2%)  44 (9.5%)  17 (3.7%)  38 (8.2%)  44 (9.5%)  48 (10.4%)  $  40 (8.7%)  37 (8.0%)  11 (2.4%)  10 (2.2%)  $  57 (12.3%) | 78 (17.5%)  63 (14.1%)  38 (8.5%)  33 (7.4%)  39 (8.7%)  34 (7.6%)  11 (2.5%)  48 (10.8%)  26 (5.8%)  9 (2.0%)  25 (5.6%)  $  40 (9.0%) | 49 (21.9%)  22 (9.8%)  13 (5.8%)  11 (4.9%)  12 (5.4%)  16 (7.1%)  $  27 (12.0%)  9 (4.0%)  8 (3.6%)  12 (5.4%)  $  38 (17.0%) | <0.001 |

* Also includes pharmacists, healthcare scientists, ambulance workers and those in optical roles.

† Work areas are binary ‘dummy’ variables which compares all those who selected an area (from a non-mutually exclusive list) to those that did not. Therefore there are different amounts of missing data for each dummy variable and this is presented as a range.

Percentages are computed column-wise . P values are from chi-squared tests for categorical variables and Kruskal-Wallis tests for continuous variables.

IQR – interquartile range; med – median; NA – nursing associate; PPE – personal protective equipment;
