# Supplementary material for: Risk factors associated with SARS-CoV-2 infection in a multiethnic cohort of United Kingdom healthcare workers (UK-REACH): A cross-sectional analysis
Source: PLoS Med. 2022 May 26;19(5):e1004015. doi: 10.1371/journal.pmed.1004015 (PMC9187071; doi:10.1371/journal.pmed.1004015)
Supplement: S4 Table — CI, confidence interval; OR, odds ratio; Ref, reference category for categorical variable. (DOCX) [file pmed.1004015.s006.docx]

**S4 Table. Description of cohort, including those not working during lockdown, by infection status**

| **Variable** | **Not infected**  **9750 (77.7%)** | **Infected**  **2791 (22.3%)** | **Unadjusted OR (95% CI)** | **P value** |
| --- | --- | --- | --- | --- |
| **Age**, med(IQR) | 45 (35 – 55) | 42 (32 – 52) | 0.84 (0.81 – 0.87) | <0.001 |
| **Sex**  Male  Female | 2289 (23.5%)  7441 (76.5%) | 688 (24.7%)  2094 (75.3%) | Ref  0.94 (0.85 – 1.03) | -  0.20 |
| **Ethnicity**  White  Asian  Black  Mixed  Other | 6883 (70.6%)  1856 (19.0%)  391 (4.0%)  417 (4.3%)  203 (2.1%) | 1912 (68.5%)  562 (20.1%)  144 (5.2%)  112 (4.0%)  61 (2.2%) | Ref  1.09 (0.98 – 1.21)  1.33 (1.09 – 1.62)  0.97 (0.78 – 1.20)  1.08 (0.81 – 1.45) | -  0.12  0.005  0.76  0.60 |
| **Migration status**  Born in UK  Born abroad | 7188 (73.9%)  2536 (26.1%) | 1983 (71.1%)  805 (28.9%) | Ref  1.15 (1.05 – 1.26) | -  0.003 |
| **Religiosity**  Not religious  Fairly important  Very important  Extremely important | 5549 (58.3%)  2049 (21.5%)  942 (9.9%)  985 (10.3%) | 1494 (54.8%)  588 (21.6%)  296 (10.9%)  350 (12.8%) | Ref  1.06 (0.95 – 1.18)  1.16 (1.01 – 1.34)  1.31 (1.14 – 1.50) | -  0.26  0.04  <0.001 |
| **Household size,** med (IQR) | 2 (1 – 3) | 2 (1 – 3) | 1.05 (1.02 – 1.08) | 0.002 |
| **Cohabitation**  Does not live with other key workers  Lives with other key workers | 5255 (54.2%)  4433 (45.8%) | 1319 (47.5%)  1459 (52.5%) | Ref  1.31 (1.21 – 1.43) | -  <0.001 |
| **Accommodation**  Does not have shared spaces  Has shared spaces | 8019 (82.7%)  1673 (17.3%) | 2229 (80.3%)  548 (19.7%) | Ref  1.18 (1.06 – 1.31) | -  0.003 |
| **IMD**  1 (most deprived)  2  3  4  5 (least deprived) | 806 (9.4%)  1376 (16.1%)  1796 (21.0%)  2087 (24.4%)  2477 (29.0%) | 306 (12.0%)  464 (18.2%)  505 (19.8%)  584 (22.9%)  687 (27.0%) | 1.31 (1.11 – 1.54)  1.19 (1.03 – 1.38)  Ref  0.98 (0.86 – 1.13)  0.97 (0.85 – 1.11) | 0.001  0.02  -  0.80  0.64 |
| **Social mixing**  None or all remote  Face to face (with SD)  Physical contact | 2402 (24.7%)  6044 (62.3%)  1262 (13.0%) | 763 (27.5%)  1603 (57.7%)  413 (14.9%) | Ref  0.83 (0.76 – 0.92)  1.03 (0.90 – 1.18) | -  <0.001  0.67 |
| **Comorbidities**  Not diabetic  Diabetic | 8963 (96.0%)  373 (4.0%) | 2555 (96.0%)  106 (4.0%) | Ref  1.00 (0.80 – 1.24) | -  0.98 |
| **Comorbidities**  Not immunosuppressed  Immunosuppressed | 8993 (96.3%)  343 (3.7%) | 2584 (97.1%)  77 (2.9%) | Ref  0.79 (0.62 – 1.02) | -  0.08 |
| **Shielding status**  Not advised to shield  Advised to shield | 9260 (95.3%)  454 (4.7%) | 2676 (96.4%)  100 (3.6%) | Ref  0.77 (0.62 – 0.96) | -  0.02 |
| **Smoking status**  Never/ex-smoker  Current smoker | 9152 (94.7%)  510 (5.3%) | 2663 (96.4%)  99 (3.6%) | Ref  0.67 (0.53 – 0.83) | -  <0.001 |
| **COVID-19 vaccination status (at the time of questionnaire completion)**  Unvaccinated  Vaccinated | 2667 (37.8%)  4381 (62.1%) | 829 (42.3%)  1129 (57.7%) | Ref  0.83 (0.75 – 0.92) | -  <0.001 |
| **Work region**  West Midlands  London  South East England  South West England  East of England  East Midlands  North East England  North West England  Yorkshire and the Humber  Wales  Scotland  Northern Ireland | 743 (8.5%)  1225 (14.0%)  1177 (13.5%)  843 (9.7%)  742 (8.5%)  977 (11.2%)  382 (4.4%)  906 (10.4%)  681 (7.8%)  290 (3.3%)  627 (7.2%)  130 (1.5%) | 230 (9.3%)  432 (17.5%)  308 (12.5%)  156 (6.3%)  183 (7.4%)  256 (10.4%)  113 (4.6%)  353 (14.3%)  233 (9.4%)  99 (4.0%)  91 (3.7%)  20 (0.8%) | Ref  1.14 (0.95 – 1.36)  0.84 (0.69 – 1.02)  0.60 (0.48 – 0.75)  0.80 (0.64 – 0.99)  0.84 (0.69 – 1.03)  0.94 (0.73 – 1.22)  1.25 (1.02 – 1.53)  1.10 (0.89 – 1.36)  1.10 (0.82 – 1.44)  0.47 (0.36 – 0.61)  0.52 (0.32 – 0.83) | -  0.16  0.07  <0.001  0.04  0.10  0.66  0.03  0.39  0.57  <0.001  0.007 |

* Also includes pharmacists, healthcare scientists, ambulance workers and those in optical roles.

Percentages are computed column-wise other than the total of infected and non-infected HCWs which are computed row-wise.

95%CI – 95% confidence interval, OR – odds ratio, Ref – reference category for categorical variable
