# Supplementary material for: Risk factors associated with SARS-CoV-2 infection in a multiethnic cohort of United Kingdom healthcare workers (UK-REACH): A cross-sectional analysis
Source: PLoS Med. 2022 May 26;19(5):e1004015. doi: 10.1371/journal.pmed.1004015 (PMC9187071; doi:10.1371/journal.pmed.1004015)
Supplement: S6 Table — aOR, adjusted odds ratio; PPE, personal protective equipment; Ref, reference category for categorical variables; SARS-CoV-2, Severe Acute Respiratory Syndrome Coronavirus 2. (DOCX) [file pmed.1004015.s008.docx]

**S6 Table. Multivariable analysis of factors associated with SARS-CoV-2 infection in complete cases**

| Variable | WITH COVID-19 VACCINATION VARIABLE | | | |
| --- | --- | --- | --- | --- |
|  | Adjusted for demographic, home and work factors during lockdown (n=4,383) | | Adjusted for demographic and home factors (n=4,645) | |
|  | aOR (95% CI) | p value | aOR (95% CI) | p value |
| **Demographic and household factors** | | | | |
| **Age*** | 0.95 (0.88 – 1.02) | 0.18 | 0.84 (0.79 – 0.90) | <0.001 |
| **Sex**  Male  Female | Ref  1.07 (0.89 – 1.28) | -  0.48 | Ref  0.90 (0.76 – 1.06) | -  0.20 |
| **Ethnicity**  White  Asian  Black  Mixed  Other | Ref  0.93 (0.74 – 1.17)  1.02 (0.70 – 1.49)  0.95 (0.66 – 1.36)  0.92 (0.54 – 1.57) | -  0.53  0.91  0.76  0.77 | Ref  0.96 (0.78 – 1.18)  0.98 (0.70 – 1.39)  1.01 (0.73 – 1.40)  0.89 (0.56 – 1.42) | -  0.70  0.93  0.95  0.62 |
| **Migration status**  Born in UK  Born abroad | Ref  1.20 (0.99 – 1.46) | -  0.07 | Ref  1.18 (0.99 – 1.42) | -  0.07 |
| **Religiosity**  Not important or not religious  Fairly important  Very important  Extremely important | Ref  1.00 (0.83 – 1.21)  0.88 (0.67 – 1.15)  1.13 (0.88 – 1.46) | -  0.98  0.35  0.35 | Ref  0.97 (0.80 – 1.17)  1.06 (0.82 – 1.36)  1.14 (0.90 – 1.44) | -  0.73  0.65  0.29 |
| **Index of multiple deprivation**  1 (most deprived)  2  3  4  5 (least deprived) | 1.03 (0.77 – 1.38)  1.09 (0.85 – 1.38)  Ref  0.95 (0.76 – 1.19)  1.02 (0.82 – 1.27) | 0.82  0.50  -  0.65  0.85 | 1.09 (0.83 – 1.44)  1.03 (0.82 – 1.30)  0.83 (0.67 – 1.02)  0.89 (0.72 – 1.09) | 0.52  0.82  0.08  0.26 |
| **Household size** | 1.00 (0.94 – 1.06) | 0.89 | 1.02 (0.96 – 1.08) | 0.53 |
| **Cohabitation**  Does not live with other key workers  Lives with other key workers | Ref  1.17 (1.00 – 1.37) | -  0.04 | Ref  1.30 (1.12 – 1.51) | -  0.001 |
| **Accommodation**  Does not have shared spaces  Has shared spaces | Ref  0.98 (0.79 – 1.20) | -  0.82 | Ref  1.02 (0.84 – 1.23) | -  0.85 |
| **Social mixing with others outside household**  None / remote only  Face to face with social distancing  With physical contact | Ref  0.99 (0.83 – 1.17)  0.95 (0.74 – 1.24) | -  0.87  0.74 | Ref  0.99 (0.84 – 1.16)  0.99 (0.77 – 1.27) | -  0.89  0.95 |
| **Comorbidities**  Diabetes  Immunosuppression | 1.18 (0.80 – 1.76)  0.77 (0.45 – 1.30) | 0.40  0.32 | 1.34 (0.95 – 1.91)  0.80 (0.49 – 1.32) | 0.10  0.39 |
| **Shielding status**  Not advised to shield  Advised to shield | Ref  1.37 (0.88 – 2.11) | -  0.16 | Ref  1.20 (0.82 – 1.77) | -  0.35 |
| **Smoking status**  Ex or non-smoker  Current smoker | Ref  0.61 (0.41 – 0.90) | -  0.01 | Ref  0.71 (0.49 – 1.03) | -  0.01 |
| **COVID-19 vaccination status (at the time of questionnaire completion)**  Unvaccinated  Vaccinated | Ref  0.58 (0.49 – 0.70) | -  <0.001 | Ref  0.83 (0.71 – 0.96) | -  0.01 |
| **Region of workplace†**  West Midlands  London  South East England  South West England or Channel Islands  East of England  East Midlands  North East England  North West England or Isle of Man  Yorkshire and the Humber  Wales, Scotland or Northern Ireland | Ref  0.82 (0.63 – 1.06)  0.59 (0.43 – 0.81)  0.68 (0.50 – 0.92)  0.78 (0.57 – 1.06)  0.86 (0.64 – 1.16)  0.86 (0.60 – 1.23)  1.07 (0.81 – 1.40)  1.08 (0.79 – 1.47)  1.04 (0.41 – 2.60) | -  0.13  0.001  0.01  0.12  0.32  0.42  0.64  0.62  0.94 | Ref  1.16 (0.88 – 1.53)  0.81 (0.60 – 1.08)  0.66 (0.47 – 0.93)  0.83 (0.60 – 1.15)  0.95 (0.65 – 1.38)  1.35 (1.00 – 1.81)  1.10 (0.79 – 1.52)  1.10 (0.79 – 1.53)  0.85 (0.34 – 2.12) | -  0.30  0.15  0.02  0.26  0.74  0.80  0.05  0.56  0.72 |
| **Time between questionnaire rollout and questionnaire completion (per day)** | 1.01 (1.01 – 1.01) | <0.001 | 1.00 (1.00 – 1.01) | <0.001 |
| **Occupational factors** | | | | |
| **Occupation**  Doctor or medical support  Nurse, nursing associate or Midwife  Allied health professional ^‡^  Dental  Admin, estates or other | Ref  1.36 (1.07 – 1.73)  1.01 (0.81 – 1.25)  0.70 (0.45 – 1.08)  1.40 (0.87 – 2.25) | -  0.01  0.93  0.11  0.17 | -  -  -  -  - | -  -  -  -  - |
| **Transport to work**  Alone or with members of household  With others outside household | Ref  1.10 (0.85 – 1.41) | -  0.47 | -  - | -  - |
| **Number of SARS-CoV-2 positive patients attended to per week (with physical contact)**  None  1 – 5  6 – 20  ≥ 21 | Ref  1.67 (1.35 – 2.06)  2.32 (1.81 – 2.96)  3.07 (2.24 – 4.22) | -  <0.001  <0.001  <0.001 | -  -  -  - | -  -  -  - |
| **Access to appropriate PPE**  Not applicable or all/most the time  Some of the time or less frequently | Ref  1.34 (1.15 – 1.57) | -  <0.001 | -  - | -  - |
| **Aerosol generating procedure exposure**  Less than weekly exposure  At least weekly exposure | Ref  0.90 (0.73 – 1.11) | -  0.33 | -  - | -  - |
| **Night shift pattern**  Never works nights  Works nights less than weekly  Works nights weekly or always | Ref  1.08 (0.87 – 1.34)  0.86 (0.66 – 1.13) | -  0.47  0.28 | -  -  - | -  -  - |
| **Work areas**  Ambulance  Community clinical setting /primary care  Non clinical community setting  Emergency Department  Intensive Care Unit  Hospital Inpatient  Hospital Outpatient  Hospital non-clinical area or laboratory  Psychiatric hospital  Maternity  Nursing or Care Home  University  Home | 1.97 (1.28 – 3.04)  0.96 (0.78 – 1.18)  0.89 (0.60 – 1.31)  1.03 (0.81 – 1.32)  0.75 (0.56 – 0.99)  1.56 (1.29 – 1.89)  0.96 (0.78 – 1.19)  0.79 (0.52 – 1.22)  0.93 (0.71 – 1.22)  1.02 (0.60 – 1.74)  1.12 (0.70 – 1.80)  0.86 (0.48 – 1.52)  0.78 (0.62 – 0.98) | 0.002  0.70  0.55  0.80  0.04  <0.001  0.74  0.30  0.59  0.93  0.63  0.60  0.04 | -  -  -  -  -  -  -  -  -  -  -  -  - | -  -  -  -  -  -  -  -  -  -  -  -  - |

Supplementary Table 6 shows the results of two multivariable logistic regression analyses analysing only those with no missing data in any variable of interest. These analyses examine the association of demographic and household factors with infection (in the larger cohort), and the other is additionally adjusted for occupational factors (in those working during lockdown).

*for each decade increase in age. † Wales, Scotland and Northern Ireland have been combined for this sensitivity analysis. This is because English IMD categories were imputed for those living in these nations. Therefore very few of those working in Scotland, Wales or Northern Ireland are included in this complete case analysis necessitating collapse of these levels of the work region variable into one level. ‡ Also includes pharmacists, healthcare scientists, ambulance workers and those in optical roles.

Analyses adjusted for all other variables in the table (with the exception of the exclusion of occupational predictors in the right hand columns – as indicated by the lack of results in the relevant sections).

All occupational factors (other than region of workplace) relate to work circumstances during the weeks following the first UK national lockdown on March 23^rd^ 2020. When asked about work areas participants could select multiple answers , therefore the work areas variables are ‘dummy’ variables comparing all those that did not select an area (reference) with all those that did. Region of workplace is included in the analysis of household and demographic factors as a proxy for the participants region of residence.

aOR – adjusted odds ratio, PPE – personal protective equipment, Ref – reference category for categorical variables, SARS-CoV-2 – severe acute respiratory syndrome coronavirus-2
