# Supplementary material for: Risk factors associated with SARS-CoV-2 infection in a multiethnic cohort of United Kingdom healthcare workers (UK-REACH): A cross-sectional analysis
Source: PLoS Med. 2022 May 26;19(5):e1004015. doi: 10.1371/journal.pmed.1004015 (PMC9187071; doi:10.1371/journal.pmed.1004015)
Supplement: S8 Table — aOR, adjusted odds ratio; PPE, personal protective equipment; Ref, reference category for categorical variables; SARS-CoV-2, Severe Acute Respiratory Syndrome Coronavirus 2. (DOCX) [file pmed.1004015.s010.docx]

**S8 Table. Univariable and multivariable analysis of factors associated with SARS-CoV-2 infection using a more granular ethnicity variable in those who worked during lockdown (n=10,772)**

| Variable | Univariable | | Multivariable | |
| --- | --- | --- | --- | --- |
|  | OR (95% CI) | p value | aOR (95% CI) | p value |
| **Age*** | 0.83 (0.80 – 0.86) | <0.001 | 0.93 (0.88 – 0.97) | 0.002 |
| **Sex**  Male  Female | Ref  0.94 (0.85 – 1.04) | -  0.25 | Ref  1.03 (0.92 – 1.16) | -  0.60 |
| **Ethnicity**  White - British  White - Irish  White - Other/Gypsy Irish Traveller  Asian - Indian  Asian - Pakistani  Asian - Bangladeshi  Asian - Chinese  Asian - Other  Black - African  Black - Caribbean  Black - Other  Mixed - White & Black Caribbean  Mixed - White & Black African  Mixed - White & Asian  Mixed - Other  Arab  Other | Ref  1.29 (0.94 – 1.79)  1.21 (1.02 – 1.43)  1.00 (0.86 – 1.17)  1.66 (1.29 – 2.13)  1.28 (0.73 – 2.23)  1.02 (0.74 – 1.39)  1.26 (0.98 – 1.60)  1.60 (1.26 – 2.04)  0.99 (0.63 – 1.55)  1.77 (0.71 – 4.38)  0.74 (0.41 - 1.32)  1.03 (0.57 – 1.87)  1.15 (0.80 – 1.64)  0.83 (0.54 – 1.28)  1.13 (0.73 – 1.76)  1.11 (0.72 – 1.71) | -  0.12  0.03  0.97  <0.001  0.39  0.92  0.07  <0.001  0.97  0.22  0.30  0.92  0.45  0.41  0.57  0.64 | Ref  1.38 (0.96 – 1.97)  1.18 (0.95 – 1.47)  0.89 (0.73 – 1.08)  1.20 (0.89 – 1.62)  0.97 (0.53 – 1.77)  0.92 (0.65 – 1.30)  0.94 (0.70 – 1.26)  1.17 (0.86 – 1.58)  0.76 (0.48 – 1.23)  1.47 (0.55 – 3.90)  0.64 (0.35 – 1.17)  1.00 (0.53 – 1.89)  0.99 (0.68 – 1.45)  0.82 (0.52 – 1.30)  0.82 (0.50 – 1.35)  0.93 (0.58 – 1.50) | -  0.08  0.14  0.23  0.24  0.92  0.63  0.67  0.32  0.26  0.44  0.14  1.00  0.96  0.40  0.44  0.77 |
| **Migration status**  Born in UK  Born abroad | Ref  1.16 (1.05 – 1.29) | -  0.003 | Ref  1.01 (0.86 – 1.18) | -  0.93 |
| **Religiosity**  Not important or not religious  Fairly important  Very important  Extremely important | Ref  1.05 (0.94 – 1.18)  1.12 (0.96 – 1.31)  1.32 (1.14 – 1.53) | -  0.41  0.15  <0.001 | Ref  1.07 (0.94 – 1.21)  1.03 (0.87 – 1.23)  1.24 (1.04 – 1.46) | -  0.31  0.74  0.01 |
| **Index of multiple deprivation**  1 (most deprived)  2  3  4  5 (least deprived) | 1.22 (1.02 – 1.46)  1.17 (1.01 – 1.36)  Ref  0.99 (0.85 – 1.14)  0.94 (0.82 – 1.08) | 0.03  0.04  -  0.85  0.41 | 1.01 (0.83 – 1.23)  1.08 (0.92 – 1.26)  Ref  1.02 (0.88 – 1.19)  1.05 (0.91 – 1.21) | 0.89  0.21  -  0.76  0.48 |
| **Household size** | 1.05 (1.02 – 1.08) | 0.004 | 1.01 (0.98 – 1.05) | 0.49 |
| **Cohabitation**  Does not live with other key workers  Lives with other key workers | Ref  1.29 (1.18 – 1.41) | -  <0.001 | Ref  1.18 (1.07 – 1.30) | -  0.001 |
| **Accommodation**  Does not have shared spaces  Has shared spaces | Ref  1.17 (1.04 – 1.31) | -  0.01 | Ref  0.92 (0.81 – 1.06) | -  0.24 |
| **Social mixing with others outside household**  None / remote only  Face to face with social distancing  With physical contact | Ref  0.82 (0.74 – 0.91)  1.02 (0.89 – 1.19) | -  <0.001  0.74 | Ref  0.91 (0.81 – 1.02)  0.98 (0.84 – 1.15) | -  0.09  0.83 |
| **Comorbidities**  Diabetes  Immunosuppression | 0.96 (0.75 – 1.22)  0.75 (0.57 – 0.99) | 0.73  0.04 | 1.12 (0.87 – 1.45)  0.99 (0.72 – 1.35) | 0.37  0.93 |
| **Shielding status**  Not advised to shield  Advised to shield | Ref  0.77 (0.60 – 0.99) | -  0.05 | Ref  0.89 (0.67 – 1.18) | -  0.42 |
| **Smoking status**  Ex or non-smoker  Current smoker | Ref  0.68 (0.54 – 0.85) | -  0.001 | Ref  0.55 (0.44 – 0.71) | -  <0.001 |
| **COVID-19 vaccination status (at the time of questionnaire response)**  Unvaccinated  Vaccinated | Ref  0.77 (0.70 – 0.86) | -  <0.001 | Ref  0.62 (0.54 – 0.73) | -  <0.001 |
| **Time between questionnaire rollout and questionnaire completion (per day)** | 1.00 (1.00 – 1.00) | 0.73 | 1.01 (1.00 – 1.01) | 0.001 |
| **Occupation**  Doctor or medical support  Nurse, nursing associate or Midwife  Allied health professional†  Dental  Admin, estates or other | Ref  1.14 (1.00 – 1.29)  0.88 (0.78 – 0.98)  0.50 (0.38 – 0.67)  0.68 (0.54 – 0.86) | -  0.05  0.03  <0.001  0.001 | Ref  1.32 (1.13 – 1.55)  1.01 (0.88 – 1.17)  0.70 (0.51 – 0.97)  1.19 (0.91 – 1.54) | -  0.001  0.85  0.03  0.20 |
| **Transport to work**  Alone or with members of household  With others outside household | Ref  1.44 (1.25 – 1.66) | -  <0.001 | Ref  1.09 (0.93 – 1.28) | -  0.30 |
| **Number of SARS-CoV-2 positive patients attended to per week (with physical contact)**  None  1 – 5  6 – 20  ≥ 21 | Ref  2.11 (1.88 – 2.36)  2.83 (2.49 – 3.20)  3.43 (2.90 – 4.05) | -  <0.001  <0.001  <0.001 | Ref  1.70 (1.49 – 1.95)  2.14 (1.83 – 2.51)  2.59 (2.10 – 3.18) | -  <0.001  <0.001  <0.001 |
| **Access to appropriate PPE**  Not applicable or all/most the time  Some of the time or less frequently | Ref  1.54 (1.41 – 1.70) | -  <0.001 | Ref  1.29 (1.17 – 1.43) | -  <0.001 |
| **Aerosol generating procedure exposure**  Less than weekly exposure  At least weekly exposure | Ref  1.48 (1.33 – 1.64) | -  <0.001 | Ref  0.91 (0.80 – 1.04) | -  0.18 |
| **Night shift pattern**  Never works nights  Works nights less than weekly  Works nights weekly or always | Ref  1.81 (1.61 – 2.03)  1.54 (1.35 – 1.76) | -  <0.001  <0.001 | Ref  1.10 (0.96 – 1.27)  0.85 (0.72 – 1.00) | -  0.18  0.05 |
| **Work areas**  Ambulance  Community clinical setting / primary care  Non clinical community setting  Emergency Department  Intensive Care Unit  Hospital Inpatient  Hospital Outpatient  Hospital non-clinical area or laboratory  Psychiatric hospital  Maternity  Nursing or Care Home  University  Home | 2.21 (1.80 – 2.72)  0.68 (0.61 – 0.76)  0.70 (0.56 – 0.87)  1.74 (1.51 – 2.00)  1.25 (1.07 – 1.46)  1.99 (1.80 – 2.19)  1.01 (0.90 – 1.14)  0.68 (0.58 – 0.80)  1.31 (1.02 – 1.69)  0.78 (0.59 – 1.02)  1.32 (1.00 – 1.75)  0.80 (0.57 – 1.11)  0.57 (0.50 – 0.66) | <0.001  <0.001  0.001  <0.001  0.004  <0.001  0.86  <0.001  0.03  0.07  0.05  0.18  <0.001 | 2.01 (1.56 – 2.58)  0.93 (0.81 – 1.06)  0.91 (0.72 – 1.16)  1.10 (0.93 – 1.29)  0.76 (0.64 – 0.92)  1.55 (1.37 – 1.75)  0.94 (0.82 – 1.08)  0.86 (0.73 – 1.04)  1.31 (1.00 – 1.71)  0.67 (0.50 – 0.89)  1.35 (1.00 – 1.82)  0.90 (0.63 – 1.28)  0.79 (0.68 – 0.92) | <0.001  0.27  0.45  0.27  0.004  <0.001  0.37  0.12  0.05  0.006  0.05  0.56  0.002 |
| **Work region**  West Midlands  London  South East England  South West England  East of England  East Midlands  North East England  North West England  Yorkshire and the Humber  Wales  Scotland  Northern Ireland | Ref  1.12 (0.92 – 1.37)  0.85 (0.69 – 1.04)  0.56 (0.45 – 0.72)  0.82 (0.65 – 1.03)  0.79 (0.64 – 0.98)  0.90 (0.69 – 1.18)  1.28 (1.05 – 1.57)  1.10 (0.88 – 1.37)  1.09 (0.81 – 1.46)  0.44 (0.33 – 0.58)  0.49 (0.29 – 0.82) | -  0.25  0.12  <0.001  0.09  0.03  0.44  0.01  0.41  0.58  <0.001  0.007 | Ref  1.10 (0.88 – 1.37)  0.84 (0.68 – 1.05)  0.57 (0.45 – 0.73)  0.76 (0.59 – 1.96)  0.87 (0.70 – 1.09)  0.86 (0.65 – 1.15)  1.21 (0.98 – 1.49)  1.12 (0.88 – 1.42)  1.10 (0.81 – 1.49)  0.43 (0.32 – 0.59)  0.44 (0.26 – 0.76) | -  0.39  0.12  <0.001  0.03  0.24  0.31  0.08  0.36  0.54  <0.001  0.003 |

Supplementary Table 5 shows the results of univariable and multivariable logistic regression analyses, examining the association of covariates with infection, in the cohort working during lockdown using the office or national statistics’ 18 ethnicity categories (with gypsy/Irish traveller collapsed into ‘White – Other’ due to low numbers of respondents in the former).

*for each decade increase in age. † Also includes pharmacists, healthcare scientists, ambulance workers and those in optical roles.

Analyses adjusted for all other variables in the table.

All occupational factors (other than region of workplace) relate to work circumstances during the weeks following the first UK national lockdown on March 23^rd^ 2020. When asked about work areas participants could select multiple answers , therefore the work areas variables are ‘dummy’ variables comparing all those that did not select an area (reference) with all those that did. Region of workplace is included in the analysis of household and demographic factors as a proxy for the participants region of residence.

aOR – adjusted odds ratio, PPE – personal protective equipment, Ref – reference category for categorical variables, SARS-CoV-2 – severe acute respiratory syndrome coronavirus-2
