# Supplementary material for: Risk factors associated with SARS-CoV-2 infection in a multiethnic cohort of United Kingdom healthcare workers (UK-REACH): A cross-sectional analysis
Source: PLoS Med. 2022 May 26;19(5):e1004015. doi: 10.1371/journal.pmed.1004015 (PMC9187071; doi:10.1371/journal.pmed.1004015)
Supplement: Attachment — Submitted filename: UK-REACH infection rebuttal letter 2.docx [file pmed.1004015.s011.docx]

**­­­**

**Response to Editor’s comments**

**Comment 1: At this stage, we ask that you include a short, non-technical Author Summary of your research to make findings accessible to a wide audience that includes both scientists and non-scientists. The Author Summary should immediately follow the Abstract in your revised manuscript. This text is subject to editorial change and should be distinct from the scientific abstract. Please see our author guidelines for more information: [link to author guidelines]**

*Response: We have now included a non-technical author summary after the abstract in our revised manuscript. This reads as follows:*

***“Why was this study done?***

- *Previous research has shown that healthcare workers, particularly those from ethnic minority groups, are at high risk of COVID-19.*
- *This study aims to provide information about the reasons why these groups face a high risk of COVID-19.*

***What did the researchers do and find?***

- *We conducted an electronic survey of over 12,000 UK healthcare workers (30% of whom were from ethnic minority groups) as part of the UK-REACH study, in order to gather information about home and work factors that might be associated with COVID-19.*
- *Home factors associated with a higher risk of infection included younger age and living with other ‘key workers’. Occupational factors associated with a higher risk of infection included attending to higher numbers of COVID-19 patients, working in a nursing or midwifery role, reporting a lack of access to appropriate personal protective equipment (PPE) and working in hospital inpatient and ambulance settings.*
- *Healthcare workers from certain ethnic minority groups were at higher risk of COVID-19 than White healthcare workers. There are differences in home and occupational factors that affect COVID-19 risk between ethnic groups.*

***What do these findings mean?***

- *We have identified key risk factors for COVID-19 in UK healthcare workers and have demonstrated that ethnic groups differ in home and work factors that affect COVID-19 risk. These factors may therefore explain the high risk of COVID-19 faced by ethnic minority healthcare workers.*
- *Our findings can be used to identify and protect ‘at risk’ healthcare workers as the pandemic continues.*
- *As with all studies of this kind, caution is required when interpreting our findings as our results may be influenced by the differences between the group of healthcare workers who completed our survey compared to the healthcare workforce as a whole.”*

**Comment 2: Please define healthcare worker (HCW) in the legend for Figure 1.**

*Response: We have added the following text to the legend for Figure 1.*

*“HCW – Healthcare worker (those in professional healthcare roles or ancillary workers in a healthcare setting or registered with one of the seven participating UK healthcare professional regulatory bodies – see methods for a list of participating regulatory bodies).”*

**Comment 3. Please ensure all Supporting Information items are cited in-text using the format outlined here: [link to guidelines for preparation of supporting information]**

*Response: We have followed the guidelines for citation of supporting information items. In doing so, we have included a section titled “Supporting information captions” at the end of the manuscript. We have also uploaded our supporting information files as separate files labelled S1 Table through to S8 Table.*

**Comment 4. Related to the above, please ensure you cite the CHERRIES checklist in your Supporting Information in a similar manner to how you have cited your STROBE checklist (lines 108-109).**

*Response: We have now moved the citation of the CHERRIES checklist to earlier in the methods. This directly follows the citation of the STROBE checklist. This section reads as follows:*

*“This study is reported as per the Strengthening the Reporting of Observational Studies in Epidemiology (STROBE) guideline (S1 Checklist) and the Checklist for Reporting Results of Internet E-Surveys (CHERRIES) (S2 Checklist)[1, 2].”*

**Response to Reviewer comments**

**Reviewer 1.

Comment 1. We thank the authors for addressing our previous comments, in particular the reanalysis including vaccination status, which as perhaps might be expected was a fairly important factor.**

*Response: We thank Reviewer 1 for their expert opinion on our original and revised manuscript. Their comments, particularly those related to the inclusion of vaccination status as a covariate, have markedly improved the manuscript.*

**Comment 2. A couple of points might be considered:**

*Response: Thank you for these further comments. We will address each one in turn below.*

**Comment 3. While the lowered infection risk for Scotland and South West England has been discussed (Line 428 onwards), Northern Ireland appears to have a similarly reduced risk too, compared to the reference region (West Midlands). This might be discussed too if possible.**

*Response: We have now added to the discussion to indicate that the same points raised in relation to Scotland and South West England, which relate to differences in population density, proportion of the population from ethnic minority groups and government imposed restrictions to control the spread of COVID-19, might also contribute to the reduced risk of COVID-19 for HCWs in Northern Ireland, as compared to those in the West Midlands. This section of the discussion now reads:*

*“Risk of infection differed by UK region of workplace with HCWs in South West England, Scotland and Northern Ireland being at lower risk than those working in the West Midlands. Compared to the West Midlands, these areas have a lower population density and a lower proportion of the population are from ethnic minority groups [3-6], factors associated with a decreased risk of SARS-CoV-2 transmission[7, 8]. Additionally, government imposed restrictions aimed at slowing viral transmission differed between the UK nations and this may have influenced the lower infection risk seen amongst those working in Scotland and Northern Ireland[7].”*

**Comment 4. In Table 3, for "Access to appropriate PPE", it might be considered as to whether combining "Not applicable" and "all/most of the time" to be appropriate.**

*Response: Reviewer 1 is correct, we combined the responses “Not applicable” with “all/most of the time” for the variable relating to access to personal protective equipment (PPE). We made this decision for two reasons. Firstly, we did not want to exclude those who did not require PPE to perform their job role. Secondly, we felt that, had we included a three level variable with a “not applicable” category then this would have overlapped considerably with those indicating that they did not have contact with COVID-19 patients and could have introduced problems relating to collinearity in both the imputation models and the regression models used in the analysis.*

*Changing the coding of this variable would necessitate a further round of imputation and analysis and introduce a significant delay in revising the manuscript. It is likely this would be for limited gain (and for reasons outlined above, may actually detract from the results). We would therefore respectfully request that the coding of this variable remain in its current form.*

**Reviewer 3.**

**Comment 1. Thank you for your excellent response. All of my comments have been addressed appropiately. With best wishes Silvan Liche**

*Response: We are extremely grateful to Reviewer 3 for providing their expert opinion on our manuscript. We feel our work has vastly improved as a result and we hope that it will now prove suitable for publication.*

**References**

1.	Equator Network. Improving the quality of Web surveys: the Checklist forReporting Results of Internet E-Surveys (CHERRIES) 2013 [cited 2021 21st June]. Available from: <https://www.equator-network.org/reporting-guidelines/improving-the-quality-of-web-surveys-the-checklist-for-reporting-results-of-internet-e-surveys-cherries/>.

2.	Eysenbach G. Improving the quality of Web surveys: the Checklist for Reporting Results of Internet E-Surveys (CHERRIES). J Med Internet Res. 2004;6(3):e34-e. doi: 10.2196/jmir.6.3.e34. PubMed PMID: 15471760.

3.	Office for National Statistics. Estimates of the population for the UK, England and Wales, Scotland and Northern Ireland 2021 [cited 2021 30th July]. Available from: <https://www.ons.gov.uk/peoplepopulationandcommunity/populationandmigration/populationestimates/datasets/populationestimatesforukenglandandwalesscotlandandnorthernireland>.

4.	UK Government. Ethnicity facts and figures. Regional ethnic diversity. 2020 [cited 2021 31st October]. Available from: <https://www.ethnicity-facts-figures.service.gov.uk/uk-population-by-ethnicity/national-and-regional-populations/regional-ethnic-diversity/latest#areas-of-england-and-wales-by-ethnicity>.

5.	Scotland’s Census. Ethnicity 2021 [cited 2021 31st October]. Available from: <https://www.scotlandscensus.gov.uk/census-results/at-a-glance/ethnicity/>.

6.	Northern Ireland Statistics and Research agency. Census 2011. Key Statistics for Northern Ireland. 2012 [cited 2022 4th May]. Available from: <https://www.nisra.gov.uk/sites/nisra.gov.uk/files/publications/2011-census-results-key-statistics-northern-ireland-report-11-december-2012.pdf>.

7.	Wong DWS, Li Y. Spreading of COVID-19: Density matters. PLoS One. 2020;15(12):e0242398. doi: 10.1371/journal.pone.0242398.

8.	Chadeau-Hyam M, Wang H, Eales O, Haw D, Bodinier B, Whitaker M, et al. REACT-1 study round 14: High and increasing prevalence of SARS-CoV-2 infection among school-aged children during September 2021 and vaccine effectiveness against infection in England [preprint]. medRxiv. 2021:2021.10.14.21264965. doi: 10.1101/2021.10.14.21264965.
